# Anxiety and Depression among Hypertensive Adults in Tertiary Care Hospitals of Nepal

**DOI:** 10.1155/2022/1098625

**Published:** 2022-03-11

**Authors:** Sangam Shah, Sangit Adhikari, Shova Aryal, Tara Ballav Adhikari, Sanjit Kumar Sah, Basanta Sharma Paudel, Pranil Man Singh Pradhan

**Affiliations:** ^1^Maharajgunj Medical Campus, Institute of Medicine, Tribhuvan University, Maharajgunj 44600, Nepal; ^2^Department of Public Health, Section for Global Health, Aarhus University, Aarhus 8000, Denmark; ^3^Tribhuvan University Teaching Hospital, Maharajgunj 44600, Nepal; ^4^Department of Community Medicine, Maharajgunj Medical Campus, Institute of Medicine, Tribhuvan University, Maharajgunj 44600, Nepal

## Abstract

**Introduction:**

Cooccurrence of hypertension and depression/anxiety increases the chance of cardiovascular mortality and morbidity. Therefore, this study is aimed at assessing the prevalence of anxiety and depression and their association with hypertension among hypertensive adults in a tertiary care hospital in Kathmandu, Nepal.

**Methods:**

A descriptive cross-sectional study was conducted using a semistructured self-administered questionnaire based on Hamilton Anxiety and Hamilton Depression Rating Scale. The data was entered in EPI Data and analyzed using descriptive and inferential statistics in SPSS version 22. *P* value < 0.05 was considered statistically significant.

**Results:**

A total of 260 individuals participated in the study, with a mean age of 42.6 years. About 46% of patients did not have any symptoms of depressed mood, and 73 (28.1%) of the participants experienced feelings of depressed mood only on questioning. Similarly, (151) 58.1% did not have feelings of guilt, and 48 (18.5%) participants who had the feeling of guilt had let people down. Among 260 respondents, most participants ((102) 39.2%) had mild symptoms of anxious mood, followed by (86) 33.1% participants with moderate symptoms. Only (4) 1.5% of participants had severe symptoms. Similarly, the majority of participants ((114) 43.8%) had a mild form of mental and emotional strain, followed by (72) 27.7% with moderate mental and emotional strain while (43) 16.5% had no mental and emotional strain. The occupation and marital status of the hypertensive individual was associated with anxiety and depression (*P* = ≤0.01).

**Conclusion:**

In conclusion, anxiety and depression were common among patients with hypertension. Anxiety and depression were linked to some of the patients' sociodemographic and clinical features. This study demonstrates that treating hypertension alone is not enough to improve patients' quality of life; mental illness screening among chronically ill individuals is also required.

## 1. Introduction

Noncommunicable diseases (NCDs) were responsible for 41 million deaths out of a total of 57.7 million deaths worldwide [1]. Cardiovascular diseases, diabetes, malignancies, and chronic respiratory diseases were responsible for 80% (32.8 million) of these deaths [2]. Low- and middle-income countries accounted for almost 80% of all NCD fatalities (28 million) [3]. Hypertension is responsible for over 7.5 million fatalities worldwide each year, accounting for nearly 12.8 percent of all deaths [4]. According to Kearney et al., 26.4 percent (972 million) of the world's population had hypertension in 2000 [5]. In low- and middle-income nations, hypertension is on the rise, while in developed countries, it has stayed stable or declined [6].

Nepal is currently going through an epidemiologic shift from infectious to noncommunicable diseases. The average life expectancy in Nepal has increased from 54 years in 1990 to 70 years in 2014, resulting in a double burden of noncommunicable diseases [7]. NCDs and depression have a bidirectional link, with chronic diseases increasing the risk of depression due to symptom burden, psychological stress, and functional restrictions, while depression increases the risk and severity of these NCDs [8]. This two-way link creates a vicious cycle of poor mental and physical health. Major depression can also make it challenging to manage chronic medical conditions and lead to unhealthy habits, including smoking, overeating, and a sedentary lifestyle [9].

Few studies have been undertaken in Nepal to determine the prevalence and relationship between depression and anxiety in patients with hypertension [10, 11]. The available data show a wide range of reported depression rates (varying from 6% to 81%) [12–14]. However, these studies were done with high-risk groups (e.g., torture survivors and refugees) or communities shortly after a conflict. Recent prevalence studies reported rates of depression (11.7% and 27.5%) in the community sample [10, 15]. Two out of every five patients in diabetes and hypertension clinics scored over locally validated depression thresholds [11, 16]. Previous studies have found a high frequency of NCD risk factors in countries like Pakistan and Iran [17–19]. Because depression and anxiety are frequently related to physical health problems in Nepal [20], a considerable proportion of patients accessing basic healthcare institutions in Nepal may be suffering from depression and/or anxiety disorder. However, no comprehensive studies to determine the prevalence of depression and anxiety among patients with hypertension seeking medical care in Nepal have yet been done.

Mental illnesses are prevalent in all countries and have a significant impact on socioeconomic development and growth [21]. At some point in their lives, more than a quarter of the world's population will suffer from a mental illness [22]. Patients with chronic illnesses like hypertension are more likely to acquire mental illnesses like anxiety and depression [23]. This link has been investigated in the past. However, the results have been mixed [24, 25]. As a result, the goal of this study was to determine the prevalence of anxiety and depression in patients with hypertension in a tertiary care center, as well as the factors that are associated to these disorders.

## 2. Methodology

### 2.1. Study Design

The study was a hospital-based descriptive cross-sectional study.

### 2.2. Study Method

This was a descriptive cross-sectional study was conducted over six months under the Department of Community Medicine, Maharajgunj Medical Campus. Prior approval was obtained from the Institutional Review Committee (IRC) of the Institute of Medicine. Written informed consent was obtained from all the study participants. Patients with a diagnosis of hypertension were included in the study. We included all the patients irrespective of the medications. Patient's demographic details, clinical presentations, and questionnaire were filled.

### 2.3. Study Site

Patients were from Tribhuvan University Teaching Hospital (TUTH) and Manmohan Cardiothoracic Vascular and Transplant Center (MCVTC) of Kathmandu valley.

### 2.4. Study Population

Adult patients diagnosed with hypertension in the outpatient department of Tribhuvan University Teaching Hospital (TUTH) and Manmohan Cardiothoracic Vascular and Transplant Center (MCVTC) were the studied population.

## 3. Sampling

### 3.1. Sampling Technique

TUTH and MCVTC hospital were purposively selected for the study. We used the nonprobability sampling technique and selected the patients diagnosed with hypertension in outpatient department (OPD) of these hospitals. The selected hypertensive patients to be fit on below criteria were eligible for our research.

#### 3.1.1. Inclusion and Exclusion Criteria

We included all the patients diagnosed with hypertension including those under the medications. Later, the patients that fit according to Joint Negotiating Committee (JNC) 7 criteria were included. We included the patients that were above 18 years of age. We excluded the patient who denied for consent.

#### 3.1.2. Sample Size

Taking prevalence of anxiety or depression among hypertensives to be 21.3% when taken in interview [26], allowable error is 5%(*d*) = 5% = 0.05.

By using formula,

(1) $$\begin{array}{c} n=\frac{Z^{2}pq}{d^{2}}, \end{array}$$

where *Z* = 1.96 at 5% level of confidence,

*p* = 21% = 0.213, 

*q* = 1 − *p* = 1 − 0.213 = 0.787.

Now,

(2) $$\begin{array}{c} n=\frac{1.96^{2}\times 0.213\times 0.787}{0.05^{2}},\\ n=257.5. \end{array}$$

Taking nonresponse rate as 10%, now, the total sample size becomes 257.5 + 10% of 257.5 = 283.33 ≈ 283.

After clearing the uncompleted form, the sample for the study was 260.

#### 3.1.3. Data Collection Tools and Techniques

Data was collected by using semistructural administrated questionnaires by the principal investigator and the team members. Blood pressure was measured with the help of sphygmomanometer and stethoscope by the trained enumerators with health science background. After 15 minutes of seated rest, 3 BP measurements were taken on the left arm. The cuff size was determined by the circumference of the upper arm. The average of the last two of the three blood pressure readings was used to calculate each participant's BP. The patients' lab data were used to determine their comorbidities. Hamilton Anxiety Scale [27] and Hamilton Depression Scale [28] were used questionnaire to assess the anxiety and depression. Hamilton Depression Scale was validated previously in Nepalese setting [29, 30] while Hamilton Anxiety Scale was not validated.

According to the Seventh Report of the Joint National Committee [31] on Prevention, Detection, Evaluation, and Treatment of High Blood Pressure (JNC 7), hypertension is classified as normal (systolic blood pressure is <120 mm of Hg and diastolic blood pressure < 80 mm of Hg), prehypertension (systolic blood pressure is 120-139 mm of Hg and diastolic blood pressure 80-89 mm of Hg), stage I hypertension (systolic blood pressure is 140-159 mm of Hg and diastolic blood pressure 90-99 mm of Hg), and stage II hypertension (systolic blood pressure is ≥160 mm of Hg and diastolic blood pressure ≥ 100 mm of Hg)

The Hamilton Depression Scale has 17 questions and total score ranging from 0-7, 8-13, 14-18, 19-22, and ≥23 is considered normal, mild, moderate, severe, and very severe depression, respectively. Similarly, the Hamilton Anxiety Scale has 14 questions and a total score ranging from 0-17, 18-24, and 25-30 is considered mild, moderate, and severe anxiety, respectively.

### 3.2. Data Management and Analysis

Data was compiled, edited, and checked daily to maintain consistency. The data was collected in Microsoft Excel (Ver. 2013). For statistical analysis, SPSS 21 (IBM Corp. Released 2012. IBM SPSS Statistics for Windows, Version 21.0. Armonk, NY: IBM Corp.) was used. Descriptive analysis was done to identify the distribution of sociodemographic characteristics of patients, and association was measured using a parametric and nonparametric test (depending upon the distribution of data). Chi-square test was applied to test the association of anxiety and depression with hypertension, gender, marital status, and occupation. The association was interpreted using the obtained *P* value from the analysis. *P* < 0.05 for the two-tailed test was considered statistically significant.

### 3.3. Ethical Approval

Ethical approval was obtained from the research ethics committee of the Institutional Review Committee (IRC) of the Institute of Medicine (IOM) (Approval number 346 (6-11E^2^)077/078). Official letters of cooperation from IRC were written to respective study districts and companies. Informed consent was obtained from all study subjects to allow the use of anonymous personal and clinical data in research. Confidentiality of the information was maintained thoroughly by deidentification

## 4. Results

### 4.1. Sociodemographic Characteristics of Participant

The mean age of study participants was 42.6 years (age range 18-92 years). Among the 260 respondents, 59.2% were female, while 40.8% were male. 68.1% (177) of patients were married, while the 28.8% (75) were unmarried, and the remaining (8) 3.1% participants were divorced. 23.5% (61) were dependent upon others with no occupation, followed by 23.1% (60) that were in service. The mean family income was Nepalese rupees 45315.4 per month. 50.8% of participants have someone in the family who has hypertension. The details of sociodemographic profile of study participants are shown in Table 1.

### 4.2. Blood Groups of the Study Participants

**Figure 1:**
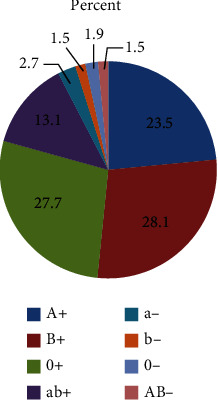
Blood groups of the study participants (*n* = 260).

There were 28.1% (73) with B positive blood group, followed by 27.7% (72) of participants with O positive blood group. The distribution of blood group of study participant is shown in Figure 1.

### 4.3. Comorbidities of the Study Participants

**Figure 2:**
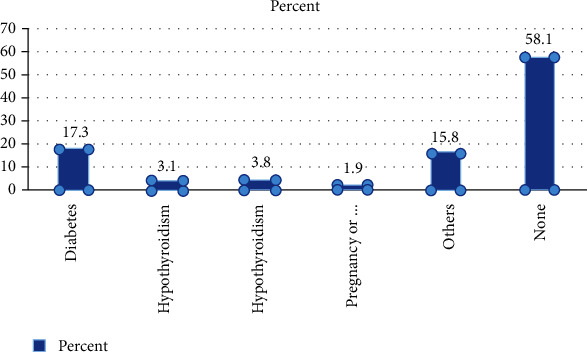
Comorbidities among the study participants (*n* = 260).

About 58.1% (151) of the participants had no other comorbidities apart from hypertension while 17.3% (45) participants had diabetes. The details of the comorbidities are shown in Figure 2.

### 4.4. Blood Pressure and Symptoms of Depression and Anxiety

The mean (SD) systolic pressure was 136.0 (16.5) mm of Hg while mean (SD) diastolic pressure was 87.7 (10.6) mm of Hg. Most of the patients ((113) 43.5%) had stage 1 hypertension while only (29) 11.2% of the patient had stage 2 hypertension. The details of the study population as hypertension category are as shown in Table 2. About 44% of the study population was under the medication of blood pressure. Overall, 80% of the participants had some degree of hypertension. This is due to the fact that patients were under regular medications for hypertension. 31.9% (83) participants of the study population had mild depression, while 6.9% (18) participants had very severe depression. 80.4% (209) patients had mild anxiety while 7.3% (19) had severe anxiety. The details of the participants having the depression and anxiety according to Hamilton Depression Rating Scale and Hamilton Anxiety Rating Scale are shown in Table 2.

About 37.2% (42) of the patients with stage I hypertension had mild depression while only (3) 10.3% of the patient with stage II hypertension had very severe depression but there was no statistically significant association between hypertension and depression (*P* = 0.692). The details of different stages of hypertension and depression are shown in Table 3. 84.1% (95) of the patients with stage I hypertension had mild anxiety while only (3) 10.3% of the patient with stage III hypertension had severe anxiety but there was no statistically significant association between hypertension and anxiety (*P* = 0.068). The details of different stages of hypertension and depression are shown in Table 3.

About 36.7% (65) of the married people with hypertension had mild depression while only 12.5% (1) of the divorced patient with hypertension had mild depression. Similarly, 88.1% (156) of the married patients had mild anxiety but no divorced patient had severe anxiety. There was a significant relation between marital status with depression (*P* = ≤0.01). The details of the marital status with anxiety and depression are shown in Table 4.

About 32.5% (50) of the female had mild depression while only (8) 7.5% of the patient had severe depression (*P* = 0.072). Similarly, about 78.6% (121) of the female patient had mild anxiety while 4.7% (5) of the male patient had severe anxiety. There was no association between gender and anxiety (*P* = 0.407). The details of the gender with anxiety and depression are shown in Table 4.

Mostly dependent 49.2% (30) hypertensive patients had mild depression while farmers with hypertension had no very severe depression. 86.9% (53) of dependent hypertensive patients had mild anxiety while only 2.8% (1) of the businessman had severe anxiety. There was a significant association between occupation and depression in a patient with hypertension (*P* = 0.001) but there was significant association between occupation and anxiety (*P* = ≤0.01). The details of the occupation with anxiety and depression in a patient with hypertension are shown in Table 5.

## 5. Discussion

In a tertiary care hospital in Nepal, anxiety and depression were common among patients with hypertension. 19.6% of the 260 patients with hypertension evaluated had moderate to severe anxiety disorders, whereas 64.2% had depressive symptoms. Patients in primary healthcare (PHC) were found to have a higher incidence of anxiety (38.4%) [32] and a similar prevalence of depression (60%) [33]. A prior study among patients in an Iranian cardiovascular outpatient clinic found similar depression [34]. The presence of a significant prevalence of mental health disorders in PHC patients with chronic conditions, as documented by the World Health Organization and the World Organization of Family Doctors [35], supports the findings of our study.

Our findings show that anxiety levels are higher in elderly patients than in younger patients. This conclusion is consistent with a recent study conducted in Malaysia among outpatients with type 2 diabetes [36]. One probable explanation for this link is that the prevalence of cardiovascular illness, stroke, and cancer is a predictor of anxiety in older chronic disease patients [37]. This study found a significant rate of depression among older patients, which is consistent with a study from a Hong Kong hypertension outpatient clinic [38]. According to the study, an increase in the prevalence of depression among older people in developing countries can be attributed to a lack of mental healthcare services and facilities, which prevents early diagnosis and treatment of depression in younger people, as well as preventing mental health progression and controlling its severity among the elderly [39]. Other factors could include their economic insecurity, which could cause delays in healthcare treatments owing to cost, as well as their limited access to healthcare. All of these factors could lead to an increase in the prevalence of depression among older people.

Females had a higher risk of anxiety and depression, according to our research. This conclusion is similar to a prior study conducted in a PHC center in Al-Khobar, Saudi Arabia [32]. This link could be explained in part by the fact that anxiety has been connected to hormonal changes associated with pregnancy, postpartum, and postmenopausal periods in women's life [40]. Another argument is that Nepalese women are more susceptible to mental illnesses as a result of a lack of mental health facilities and cultural concerns [41].

In hypertensive patients, we discovered that diabetes was linked to anxiety (*P* = 0.03). This link was discovered in a study conducted at the Supreme Council of Health's PHC clinics in Qatar [42]. Type 2 diabetes is thought to play a role in raising the development of anxiety disorders in hypertensive patients [43]. Patients with a higher number of chronic diseases to have comorbid depression-anxiety were found among patients visiting a chronic disease clinic in southwest Trinidad [44]. The notion that these findings result from people with various chronic diseases being unclear and concerned about how their lives would be affected by their illnesses appear plausible. These fears and doubts can sometimes appear as comorbid anxiety and sadness [44].

We did not assess the role of smoking as an independent risk factor for anxiety in our research. However, in a prior study [45], the link of smoking was considered an independent risk factor. Furthermore, it has been shown that anxious people are more likely to smoke [46]. A relevant study [47] indicated that smoking both reduces anxiety and appears to increase the likelihood of getting higher anxiety. Additionally, anxiety sufferers are more likely to engage in unhealthy habits such as smoking and overeating [48].

Sympathetic nervous system activity is elevated in people with significant depression, and this could be a contributing factor. Depression may also affect hypertension by increasing nonadherence to therapy for a variety of causes. For starters, favorable expectations and perceptions in the treatment's advantages and efficacy have been found to be critical for patient adherence. Patients who are depressed are likely to feel hopeless, and sticking to a treatment plan may be difficult or impossible for someone who has little faith that any effort would be worthwhile. Depressive disorder is also linked to significant functional impairment and increased healthcare utilization. Hypertension and depression may have a reciprocal link because we do not know when patients were diagnosed with hypertension. Further evidence of an association between vascular alterations associated with hypertension and depression has been found in the study of vascular depression, a kind of depression linked with numerous infarcts in the brain [49, 50]. Late-onset depression has been linked to an increased number of lesions in the brain's subcortical gray matter and deep white matter, which are thought to be caused by cerebral vascular disease [49, 50].

Psychological conditions affect hypertension. Psychosocial stressors associated with anxiety disorders raise autonomic arousal via the hypothalamic-pituitary axis, which increases circulating catecholamine, and then, this heightened arousal is associated with an increased risk of hypertension and a proinflammatory state and, consequently, development of coronary heart disease. Symptoms of depression and anxiety were found to be associated as risk factors with a diagnosis of hypertension in the population examined five years after diagnosis for depression and anxiety [51].

Some limitations in the study should be discussed. First, the sample size was limited to a single data source in the center. The study's single-center design may limit its applicability to Nepal's whole population. However, because our hospital is a referral center in Nepal, the patient pool should be regarded as reasonably large and representative, given that morbidity patterns in practice do not differ greatly across the country. The findings are consistent with those of other studies and should aid in the collection of data on this topic. Furthermore, the study's cross-sectional design makes it impossible to determine the timing of hypertension diagnosis and mental health consequences.

## 6. Conclusion

In conclusion, anxiety and depression were common among patients with hypertension. Anxiety and depression were linked to some of the patients' sociodemographic and clinical features. This study suggests that only treatment of hypertension is not an intervention for the quality of life of patients but also screening of mental illness among chronically ill patients is necessary. Additional national research is needed to develop solutions for the prevention and control of psychological distress among chronic disease patients in Nepal.

## Figures and Tables

**Table 1:** Sociodemographic characteristics of the study participants (*n* = 260).

Socio-demographic characteristics	Number (percentage)
Age mean (SD)	42.6 (17.9)
Gender	
Male	106 (40.8)
Female	154 (59.2)
Ethnicity	
Brahmin	88 (33.8)
Chhetri	77 (29.6)
Janajati	44 (16.9)
Dalit	15 (5.8)
Others	36 (13.8)
Religion	
Hindu	233 (89.60)
Buddhist	19 (7.3)
Muslim	2 (0.8)
Christian	5 (1.9)
Other	1 (0.4)
Marital status	
Married	177 (68.1)
Unmarried	75 (28.8)
Divorced	8 (3.1)
State	
State 1	36 (13.8)
State 2	36 (13.8)
Bagmati	73 (28.1)
Gandaki	32 (12.3)
Lumbini	41 (15.8)
Karnali	23 (8.8)
Sudupaschim	19 (7.3)
Occupation	
Student	54 (20.8)
Business	36 (13.8)
Service	60 (23.1)
Farmer	17 (6.5)
Dependent	61 (23.5)
Others	32 (12.3)
Family income mean (SD) (Nrs)	45315.4 (61305.3)
Education	
Primary	36 (13.8)
Secondary	36 (13.8)
Bachelor	91 (35.0)
Masters	58 (22.3)
Illiterate	39 (15.0)
Family history of hypertension	
Yes	132 (50.8)
No	128 (49.2)

Nrs: Nepalese rupee.

**Table 2:** Depression and anxiety according to Hamilton Depression Rating Scale and Hamilton Anxiety Rating Scale, respectively (*n* = 260).

Hypertension category	Number	Percent
Normal	52	20.0
Prehypertension	66	25.4
Stage 1	113	43.4
Stage 2	29	11.2
Depression		
Normal	93	35.8
Mild depression	83	31.9
Moderate depression	49	18.8
Severe depression	17	6.5
Very severe depression	18	6.9
Anxiety		
Mild	209	80.4
Moderate	32	12.3
Severe	19	7.3
Total	260	100.0

**Table 3:** Association of stages of hypertension with anxiety and depression using chi-square test (*n* = 260).

		Normal	Prehypertension	Stage I	Stag II	*P* value
Depression	Normal	22 (42.3)	25 (37.9)	40 (35.4)	6 (20.7)	0.692
	Mild	15 (28.8)	17 (25.8)	42 (37.2)	9 (31.0)	
	Moderate	9 (17.3)	13 (19.7)	19 (16.8)	8 (31.0)	
	Severe	2 (3.8)	6 (9.1)	6 (5.3)	3 (10.3)	
	Very severe	4 (7.7)	5 (7.6)	6 (5.3)	3 (10.3)	

Anxiety	Mild	45 (86.5)	51 (77.3)	95 (84.1)	18 (62.1)	0.068
	Moderate	3 (5.8)	8 (12.1)	13 (11.5)	8 (27.6)	
	Severe	4 (7.7)	7 (10.6)	5 (4.4)	3 (10.3)	

**Table 4:** Association of marital status and gender with anxiety and depression (*n* = 260).

		Married	Unmarried	Divorced	*P* value
Depression category	Normal	65 (36.7)	26 (34.7)	2 (25.0)	*P* = ≤0.01
	Mild	65 (36.7)	17 (22.7)	1 (12.5)	
	Moderate	33 (18.6)	14 (18.7)	2 (25.0)	
	Severe	10 (5.6)	5 (6.7)	2 (25.0)	
	Very severe	4 (2.3)	13 (17.3)	1 (12.5)	

Anxiety category	Mild	156 (88.1)	50 (66.7)	3 (37.5)	*P* = ≤0.01
	Moderate	13 (7.3)	14 (18.7)	5 (62.5)	
	Severe	8 (4.5)	11 (14.7)	0	
		Male	Female	*P* value	

Depression category	Normal	44 (41.5)	49 (31.8)	0.072	
	Mild	33(31.1)	50 (32.5)		
	Moderate	19 (17.9)	30 (19.5)		
	Severe	8 (7.5)	9 (5.8)		
	Very severe	2 (1.9)	16 (10.4)		
Anxiety category	Mild	88 (83.0)	121 (78.6)	0.407	
	Moderate	13 (12.3)	19 (12.3)		
	Severe	5 (4.7)	14 (9.1)		

**Table 5:** Association of occupation with anxiety and depression (*n* = 260).

		Student	Business	Service	Dependent	Others	*P* value
Depression category	Normal	22 (41.5)	12 (32.4)	27 (45.0)	11 (18.0)	21 (42.9)	*P* = ≤0.01
	Mild	11 (20.8)	15 (40.5)	17 (28.3)	30 (49.2)	10 (20.4)	
	Moderate	9 (17.0)	8 (21.6)	6 (10.0)	14 (23.0)	12 (24.5)	
	Severe	2 (3.8)	1 (2.7)	6 (10.0)	2 (3.3)	5 (10.2)	
	Very severe	9 (17.0)	1 (2.7)	4 (6.7)	4 (6.6)	1 (2.04)	

Anxiety category	Mild	36 (66.7)	32 (88.9)	50 (83.3)	53 (86.9)	38 (77.6)	*P* = ≤0.01
	Moderate	9 (16.7)	3 (8.3)	7 (11.7)	4 (6.6)	9 (18.4)	
	Severe	9 (16.7)	1 (2.8)	3 (5.0)	4 (6.6)	2 (4.1)	

## Data Availability

All the data required are in the manuscript itself. Data that are not available can be accessed from the corresponding author.

## References

[1] Noncommunicable diseases: mortality. https://www.who.int/data/gho/data/themes/topics/topic-details/GHO/ncd-mortality.

[2] World Health Organization (WHO) Noncommunicable... - Google Scholar. https://scholar.google.com/scholar_lookup?title=NoncommunicablediseaseGeneva&author=World%20HealthOrganisation.

[3] World Health Organization Projections of mortality... - Google Scholar. https://scholar.google.com/scholar_lookup?.

[4] Kilic M., Uzunçakmak T., Ede H. (2016). The effect of knowledge about hypertension on the control of high blood pressure. *International Journal of the Cardiovascular Academy*.

[5] Kearney P. M., Whelton M., Reynolds K., Muntner P., Whelton P. K., He J. (2005). Global burden of hypertension: analysis of worldwide data. *The Lancet*.

[6] Kearney P., Whelton M., Reynolds K., Whelton P. K., He J. (2004). Worldwide prevalence of hypertension: a systematic review. https://journals.lww.com/jhypertension/Fulltext/2004/01000/Worldwide_prevalence_of_hypertension___a.00003.aspx.

[7] Life expectancy at birth, total (years)|Data. https://data.worldbank.org/indicator/SP.DYN.LE00.IN.

[8] Whooley M. A., Wong J. M. (2013). Depression and cardiovascular disorders. *Annual Review of Clinical Psychology*.

[9] Katon W. J. (2003). Clinical and health services relationships between major depression, depressive symptoms, and general medical illness. *Biological Psychiatry*.

[10] Risal A., Manandhar K., Linde M., Steiner T. J., Holen A. (2016). Anxiety and depression in Nepal: prevalence, comorbidity and associations. *BMC Psychiatry*.

[11] Neupane D., Panthi B., McLachlan C. S., Mishra S. R., Kohrt B. A., Kallestrup P. (2015). Prevalence of undiagnosed depression among persons with hypertension and associated risk factors: a cross-sectional study in urban Nepal. *PLoS One*.

[12] Kohrt B., Hruschka D., Worthman C. M. (2012). Political violence and mental health in Nepal: prospective study. *The British Journal of Psychiatry*.

[13] Luitel N., Jordans M., Sapkota R. (2013). Conflict and mental health: a cross-sectional epidemiological study in Nepal. *Social Psychiatry and Psychiatric Epidemiology*.

[14] Tol W. A., Kohrt B. A., Jordans M. J. (2010). Political violence and mental health: a multi-disciplinary review of the literature on Nepal. *Social Science & Medicine*.

[15] Luitel N. P., Jordans M. J. D., Kohrt B. A., Rathod S. D., Komproe I. H. (2017). Treatment gap and barriers for mental health care: a cross-sectional community survey in Nepal. *PLoS One*.

[16] Niraula K., Kohrt B. A., Flora M. S. (2013). Prevalence of depression and associated risk factors among persons with type-2 diabetes mellitus without a prior psychiatric history: a cross-sectional study in clinical settings in urban Nepal. *BMC Psychiatry*.

[17] Esteghamati A., Meysamie A., Khalilzadeh O. (2009). Third National Surveillance of Risk Factors of Non-Communicable Diseases (SuRFNCD-2007) in Iran: methods and results on prevalence of diabetes, hypertension, obesity, central obesity, and dyslipidemia. *BMC Public Health*.

[18] Shera A. S., Rafique G., Khwaja I. A., Ara J., Baqai S., King H. (1995). Pakistan National Diabetes Survey: prevalence of glucose intolerance and associated factors in Shikarpur, Sindh Province. *Diabetic Medicine*.

[19] Safdar S., Omair A., Faisal U., Hasan H. (2004). Prevalence of hypertension in a low income settlement of Karachi, Pakistan. https://www.researchgate.net/profile/Aamir-Omair-2/publication/8175605_Prevalence_of_hypertension_in_a_low_income_settlement_of_Karachi_Pakistan/links/5846c32408aeda6968226ca2/Prevalence-of-hypertension-in-a-low-income-settlement-of-Karachi-Pakistan.pdf.

[20] Kohrt B. A. (2005). “Somatization” and “comorbidity”: a study of Jhum-Jhum and depression in rural Nepal. *Ethos*.

[21] Ambikile J. S., Iseselo M. K. (2017). Mental health care and delivery system at Temeke hospital in Dar es Salaam, Tanzania. *BMC Psychiatry*.

[22] Bacon S., Campbell T. S., Arsenault A., Lavoie K. L. (2014). The impact of mood and anxiety disorders on incident hypertension at one year. *International Journal of Hypertension*.

[23] Kessler R., Aguilar-Gaxiola S., Alonso J. (2009). The global burden of mental disorders: an update from the WHO World Mental Health (WMH) surveys. *Epidemiology and Psychiatric Sciences*.

[24] Stein D., Aguilar-Gaxiola S., Alonso J. (2014). Associations between mental disorders and subsequent onset of hypertension. *General Hospital Psychiatry*.

[25] Johannessen L., Strudsholm U., Foldager L., Munk-Jørgensen P. (2006). Increased risk of hypertension in patients with bipolar disorder and patients with anxiety compared to background population and patients with schizophrenia. *Journal of Affective Disorders*.

[26] Li Z., Li Y., Chen L., Chen P., Hu Y., Wang H. (2015). Prognostic Role of Glasgow Prognostic Score in patients with Hepatocellular Carcinoma. *Medicine*.

[27] Hamilton M. A. (1959). The assessment of anxiety states by rating. *The British Journal of Medical Psychology*.

[28] Hamilton M. (1960). A rating scale for depression. *Journal of Neurology, Neurosurgery, and Psychiatry*.

[29] Shakya R., Sitaula S., Shyangwa P. M. (2008). Depression during pregnancy in a tertiary care center of eastern Nepal. *Journal of Nepal Medical Association*.

[30] Research tools - TPO Nepal. http://www.tponepal.org/research-tools/.

[31] Das Gupta R., Bin Zaman S., Wagle K., Crispen R., Hashan M. R., Al Kibria G. M. (2019). Factors associated with hypertension among adults in Nepal as per the Joint National Committee 7 and 2017 American College of Cardiology/American Heart Association hypertension guidelines: a cross-sectional analysis of the demographic and health survey 2016. *BMJ Open*.

[32] AlKhathami A. D., Alamin M. A., Alqahtani A. M., Alsaeed W. Y., AlKhathami M. A., Al-Dhafeeri A. H. (2017). Depression and anxiety among hypertensive and diabetic primary health care patients. *Saudi Medical Journal*.

[33] Al-Qadhi W., ur Rahman S., Ferwana M. S., Abdulmajeed I. A. (2014). Adult depression screening in Saudi primary care: prevalence, instrument and cost. *BMC Psychiatry*.

[34] Bayani B., Yousefi S., Bayani M. (2011). Depression and anxiety in a cardiovascular outpatient clinic: a descriptive study. *Iranian Journal of Psychiatry*.

[35] World Health Organization Integrating mental health into primary care, a globalperspective. https://scholar.google.com/scholar_lookup?title=World.

[36] Ganasegeran K., Renganathan P., Manaf R. A., Al-Dubai S. A. R. (2014). Factors associated with anxiety and depression among type 2 diabetes outpatients in Malaysia: a descriptive cross-sectional single-centre study. *BMJ Open*.

[37] Clarke D., Currie K. C. (2009). Depression, anxiety and their relationship with chronic diseases: a review of the epidemiology, risk and treatment evidence. *Medical Journal of Australia*.

[38] Cheung B. M. Y., Au T. H. Y., Chan S. Y. (2005). The relationship between hypertension and anxiety or depression in Hong Kong Chinese. *Experimental and Clinical Cardiology*.

[39] Mahmood S., Hassan S. Z., Tabraze M. (2017). Prevalence and predictors of depression amongst hypertensive individuals in Karachi, Pakistan. *Cureus*.

[40] Russell E. J., Fawcett J. M., Mazmanian D. (2013). Risk of obsessive-compulsive disorder in pregnant and postpartum women: a meta-analysis. *The Journal of Clinical Psychiatry*.

[41] Shin S., Kim H., Liw L., Kim S. (2009). Depression and PTSD in Pashtun women in Kandahar, Afghanistan. *Asian Nursing Research*.

[42] Bener A. (2011). High prevalence of depression, anxiety and stress symptoms among diabetes mellitus patients. *The Open Psychiatry Journal*.

[43] Thomas J., Jones G., Scarinci I., Brantley P. (2003). A descriptive and comparative study of the prevalence of depressive and anxiety disorders in low-income adults with type 2 diabetes and other chronic illnesses. *Diabetes Care*.

[44] Maharaj R., Reid S., Misir A., Simeon D. T. (2005). Depression and its associated factors among patients attending chronic disease clinics in southwest Trinidad. *West Indian Medical Journal*.

[45] Lasser K., Boyd J. W., Woolhandler S., Himmelstein D. U., McCormick D., Bor D. H. (2000). Smoking and mental illness: a population-based prevalence study. *Journal of the American Medical Association*.

[46] Brown R. A., Lewinsohn P. M., Seeley J. R., Wagner E. F. (1996). Cigarette smoking, major depression, and other psychiatric disorders among adolescents. *Journal of the American Academy of Child and Adolescent Psychiatry*.

[47] Tjora T., Hetland J., Aarø L. E., Øverland S. (2011). Distal and proximal family predictors of adolescents’ smoking initiation and development: a longitudinal latent curve model analysis. *BMC Public Health*.

[48] Gonzalez J. S., Safren S. A., Cagliero E. (2007). Depression, self-care, and medication adherence in type 2 diabetes. *Diabetes Care*.

[49] Greenwald B. S., Kramer-Ginsberg E., Krishnan K. R. R. (2001). A controlled study of MRI signal hyperintensities in older depressed patients with and without hypertension. *Journal of the American Geriatrics Society*.

[50] Krishnan K. R., George L. K., Pieper C. F. (1998). Depression and social support in elderly patients with cardiac disease. *American Heart Journal*.

[51] Ginty A. T., Carroll D., Roseboom T. J., Phillips A. C., De Rooij S. R. (2013). Depression and anxiety are associated with a diagnosis of hypertension 5 years later in a cohort of late middle-aged men and women. *Journal of Human Hypertension*.

